# White Matter Integrity of the Corpus Callosum Mediates the Association Between Aging and Skin Condition

**DOI:** 10.3390/life15111664

**Published:** 2025-10-24

**Authors:** Daihaoyi Yuan, Keisuke Kokubun, Kiyotaka Nemoto, Yoshinori Yamakawa

**Affiliations:** 1 Graduate School of Economics, Kyoto University, Kyoto 606-8501, Japan; 2 Open Innovation Institute, Kyoto University, Kyoto 606-8501, Japan; kokubun.keisuke.6x@kyoto-u.jp (K.K.); yamakawa@bi-lab.org (Y.Y.); 3 Graduate School of Management, Kyoto University, Kyoto 606-8501, Japan; 4 Department of Medical Informatics and Management and Psychiatry, Institute of Medicine, University of Tsukuba, Tsukuba 305-8577, Japan; kiyotaka@nemotos.net; 5 Institute of Innovative Research, Institute of Science Tokyo, Meguro, Tokyo 226-8503, Japan; 6 ImPACT Program of Council for Science, Technology and Innovation (Cabinet Office, Government of Japan), Chiyoda, Tokyo 100-8914, Japan; 7 Office for Academic and Industrial Innovation, Kobe University, Kobe 657-0013, Japan; 8 Brain Impact, Kyoto 606-8507, Japan

**Keywords:** skin aging, white matter integrity, white matter fractional anisotropy, diffusion tensor imaging

## Abstract

This study examines whether white matter integrity mediates the link between psychological stress and skin aging. This cross-sectional study included 92 healthy Japanese adults (aged 22–62 years) who underwent diffusion tensor imaging to obtain Fractional Anisotropy Brain Healthcare Quotients (FA-BHQs) for major white matter tracts, while skin aging was assessed using Motion Scan Technology. Correlation analyses revealed significant associations among stress, skin aging, and FA-BHQ in the corpus callosum (CC) and internal capsule (IC). Mediation analyses suggested, at the statistical level, a potential that the CC fully mediates the association between stress and skin aging. These findings suggest a relationship between interhemispheric white matter integrity, psychological stress, and skin aging in line with the concept of the brain–skin axis.

## 1. Introduction

Skin aging manifests in various forms, with wrinkles and unwanted pigmentation being its most prominent characteristics [1]. During the aging process, skin undergoes a contraction of its surface, leading to the widening of the sulcus cutis and cristae cutis. The cristae cutis are the inward projections of the epidermis into the dermis at the dermal-epidermal junction, as seen histologically in vertical sections [2]. This interdigitated organization of cristae cutis and dermal papillae at the dermoepidermal interface increases the contact surface area between the epidermis and dermis, thereby enhancing dermal–epidermal adhesion. This process of widened cristae cutis thus also results in the formation of wrinkles [3]. Although photo-aging caused by UV radiation accounts for the majority of visible skin changes, recent evidence also links psychological stress to skin conditions [4,5,6].

In the brain, stress responses are mediated mainly through the hypothalamic–pituitary–adrenal (HPA) axis. Corticotropin-releasing hormone (CRH), adrenocorticotropin (ACTH), cortisol, and glucocorticoids (GC) are primarily produced as part of the stress response through the activation of the HPA axis. These stress hormones could influence skin conditions in several ways. Specifically, cortisol primarily affects the immune system by acting as an immunosuppressant [7]. CRH acts on epidermal melanocytes as a survival factor under starvation stress (anti-apoptotic) and as an inhibitor of growth factor-induced cell proliferation [8] and has been shown to exhibit pro-inflammatory effects in mast cells and keratinocytes [9,10]. ACTH also shows a pro-inflammatory effect in keratinocytes and induces differentiation in sebocytes [11,12].

Given the central role of the brain in regulating stress responses and their systemic effects on the skin, it is crucial to consider the neural pathways involved in stress processing. White matter (WM) tracts play a critical role in neural communication and in the integration of stress-related signals. Based on the existing evidence, we select seven regions of white matter [13] as regions of interest that have been implicated in the pathophysiology of anxiety, stress, and emotional disorders, including the internal capsule (IC), corpus callosum (CC), fornix, anterior corona radiata (ACR), cingulum, uncinate fasciculus (UF), and superior longitudinal fasciculus (SLF). To measure the integrity of white matter, we used the Fractional-Anisotropy Brain Healthcare Quotient (FA-BHQ), which is calculated using the FA-BHQ method developed by Nemoto et al. (2017) [14].

We selected these regions because previous studies have shown that their microstructure is associated with several emotion-related disorders, including major depressive disorder (MDD) and schizophrenia [15,16]. Specifically, the fornix, being part of the limbic system, is involved in the regulation of emotions by higher order frontal cortical brain regions [17,18]. Similarly, the UF, which connects the orbitofrontal cortex to the temporal lobe, is involved in emotional regulation, and its reduced integrity has been associated with heightened anxiety and major depressive disorder [18,19]. The cingulum, another limbic white matter tract, has also been associated with emotional processing, with structural abnormalities linked to anxiety disorders and attentional bias toward negative interpersonal stimuli [20,21]. The CC, a critical structure for interhemispheric communication, integrates cognitive and emotional processes. Alterations in its integrity have been reported in anxiety and stress-related disorders, as well as aggressive behavior in bipolar disorder [22,23]. Additionally, the anterior limb of the IC serves as a site of convergence for thalamic radiations involved in emotional processing [24]. The ACR, which connects thalamic and prefrontal regions, has been implicated in worry prediction and emotional control [25]. Also, the limbic-thalamo-cortical circuit includes thalamic projections from the IC to the prefrontal cortex. ACR functions as a part of emotion regulation systems, as a decrease in FA in ACR has been shown to be linked to bipolar I patients [26]. Finally, the SLF, a major association tract linking the parietal and frontal lobes, plays a role in attention, executive function, and emotion regulation, with reduced integrity observed in PTSD, OCD, and depression [27,28]. Table 1 summarizes the functions of regions of interest in stress and emotion regulation.

Taken together, although previous studies have highlighted the roles of stress and white matter integrity in emotional processes, it remains unclear how these factors relate to skin aging. The present study aims to address this gap by examining the associations among psychological stress, skin aging, and white matter integrity, with a focus on key tracts. By investigating the potential mediating role of white matter integrity, this study provides novel insights into the brain–skin axis, emphasizes the importance of considering neural pathways when exploring the impact of stress on skin health, and also explores possible underlying mechanisms.

To our knowledge, this is the first study to investigate the role of key white matter tracts in the relationship between skin condition—indicated by the number of cristae cutis—and stress. To examine this, we conduct an analysis to explore the mediating effect of FA-BHQ in the skin aging process.

## 2. Materials and Methods

### 2.1. Subjects

Using G*Power 3.1.9.7, the required sample size for a correlation analysis with a medium effect size (r = 0.3), 5% significance level, and 80% power was calculated as 82. This follows Cohen’s (1988) guidelines [29]. To account for a potential dropout rate of about 5% and the reduction in degrees of freedom caused by inclusion of control variables, we aimed to collect data from 90 participants. Between 24 May and 17 June 2022, 95 healthy Japanese adults were recruited from Tokyo through a smartphone application developed by an IT company for businesspeople.

**Figure 1:**
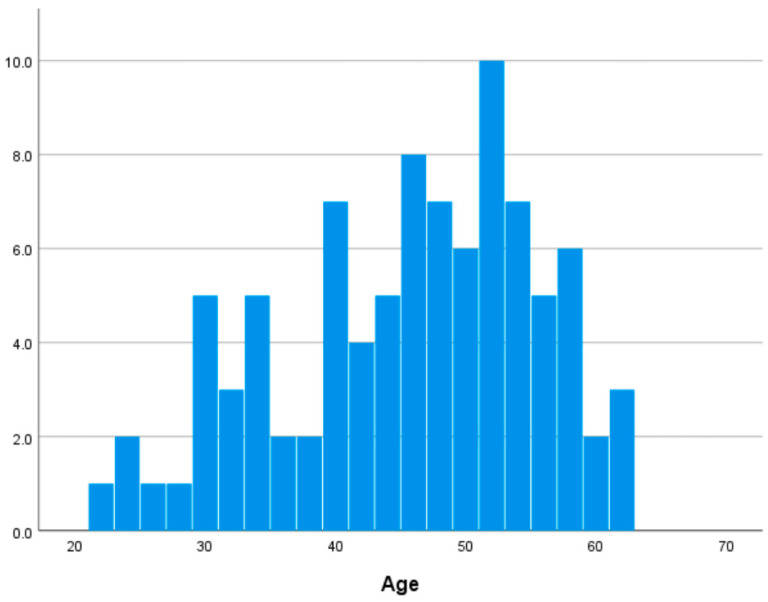
Distribution of age.

In this study, 92 participants (62 females and 30 males, aged 22–62 years, 45.076 ± 9.965 years) were included in the final analysis, excluding three individuals due to missing brain imaging data. Self-reported medical histories indicated that none of the participants had neurological, psychiatric, or other medical conditions that could influence the central nervous system. Table S3 in Supplementary Materials shows descriptive statistics of major variables. Figure 1 shows the distribution of age in our dataset.

This study was approved by the Ethics Committee of the Institute of Science Tokyo (Approval Numbers: 2022130). All procedures were conducted in accordance with relevant guidelines and regulations and the principles outlined in the Declaration of Helsinki. Written informed consent was obtained from all participants prior to their involvement, and anonymity was rigorously maintained throughout the study.

### 2.2. Skin Data

**Figure 2:**
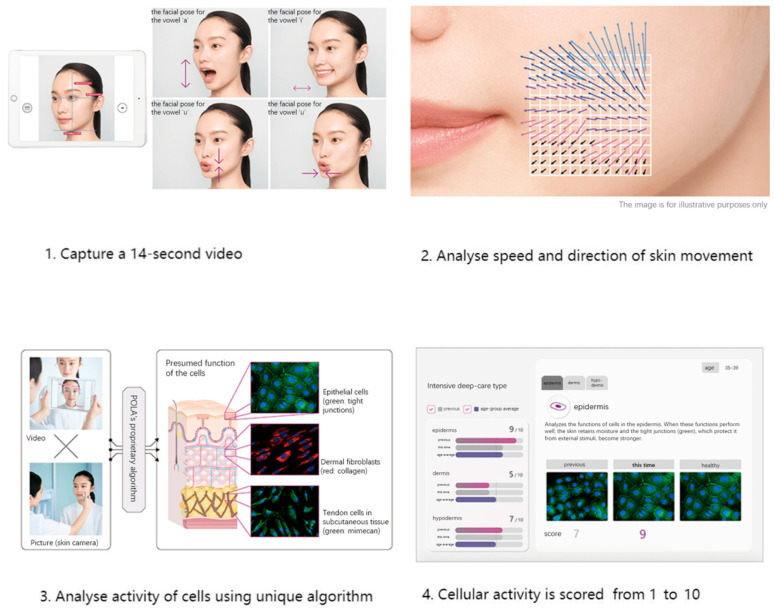
Workflow of Motion Scan Technology developed by POLA Inc.

We used skin data provided by POLA Inc. (2-2-3, Nishi-Gotanda, Shinagawa-ku, Tokyo 141-8523, Japan) [30] using Motion Scan Technology developed by POLA Inc. Figure 2 shows the detailed process of this technology. This technology has been extensively applied in research on skin aging and in the field of dermatology [31,32].

**Figure 3:**
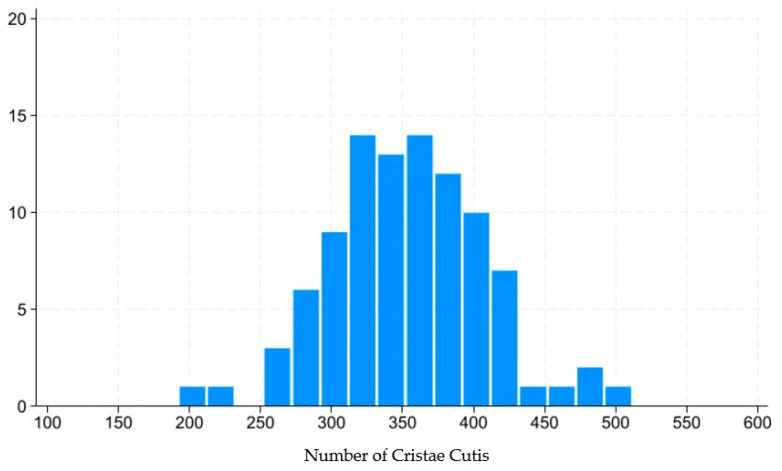
Distribution of number of cristae cutis.

This technology captures the dynamic motion of the skin’s surface to estimate the condition of the underlying skin layers, including the epidermis, dermis, and subcutaneous tissue. The system analyzes various characteristics of skin movement, such as speed and direction, extracting approximately 1.7 million data points from a 14 s video sequence. Initially, the camera aligned with the subject’s face to capture a video, which is equivalent to approximately 840 still frames. For each frame, the speed and direction of skin movement were segmented and analyzed, revealing distinctive features. POLA’s proprietary algorithm was then employed to estimate the cellular activity within the three skin layers (epidermis, dermis, and subcutaneous tissue) within approximately three minutes. The outcomes were presented on an analysis screen, where cellular activity in each layer was rated on a scale from 1 to 10. Figure 3 shows distribution of skin scale, which in this paper stands for number of cristae cutis. A higher number of cristae cutis indicates smoother skin, whereas a lower count is associated with increased wrinkles and a greater degree of skin aging. The number of cristae cutis has been shown to be closely related to skin texture and aging [33,34]. Such a method for assessing facial skin aging has also been widely employed in studies evaluating the efficacy of cosmetic formulations, as well as in investigations of skin aging and its associated environmental factors [35,36].

### 2.3. Demographic Scales

To assess demographic factors, we assessed eight variables: body mass index (BMI), sex, sleep status, sleep time, alcohol consumption, exercise, UV exposure, and stress. 

BMI in kg/m^2^ was calculated from height and weight measurements. Sleep status was categorized into 4 levels, ranging from “completely unsatisfied” to “perfectly satisfied.” Sleep time was derived from the Pittsburgh Sleep Quality Index Japanese Version (PSQI-J), based on questions about usual wake-up time, average nightly sleep duration, and difficulty falling asleep within 30 min [37,38,39]. Alcohol consumption was measured on seven levels, from “0 mL per day” to “more than 900 mL per day.” Exercise was measured using the IPAQ met-light indicator, which measures low levels of physical activity and was conducted using the International Physical Activity Questionnaire (IPAQ) Japanese Version. It was measured using MET-minute/week units, which is equivalent to kilocalories for a 60 kg person. Kilocalories were computed from MET-minutes using the following equation: MET-min x (weight in kilograms/60 kg) [40,41]. For example, the official IPAQ guidelines (IPAQ group (2005)) define physical activity intensity as 3.3, 4.0, and 8.0 METs for walking, moderate intensity, and vigorous intensity, respectively [42]. UV exposure was used to measure the extent to which participants were exposed to ultraviolet rays in their daily lives, and it was assessed with the question “Do you often get exposed to ultraviolet (UV) rays?” and categorized into four levels: “Strongly agree,” “Agree,” “Slightly agree,” and “Disagree.” Stress was assessed using the Japanese version of the Profile of Mood States 2nd Edition (POMS2), a validated instrument designed to evaluate individual mood states and generate a comprehensive mood profile [43,44,45]. POMS2 contains a summary scale called Total Mood Disturbance (TMD), which has been widely used in previous studies as an indicator of stress levels and was also employed in our research for this purpose [46,47].

Our control variables included BMI, sex, Gray Matter Brain Healthcare Quotient (GM-BHQ), sleep status, sleep time, alcohol consumption, exercise, and UV exposure. BMI was included as a control variable due to its established correlation with WM FA, as demonstrated in numerous studies [48,49]. Additionally, BMI was positively correlated with loss of skin elasticity after controlling for age [50]. Therefore, controlling for BMI is essential when examining the relationship between WM FA as well as the relationship between age and skin scale. Moreover, studies have highlighted the importance of lifestyle—such as sleep quality and duration, alcohol consumption, and physical activity or exercise—on the skin aging process [51,52]. Studies have shown that low sleep quality is associated with increased signs of intrinsic aging, and also that it is associated with being perceived as having hanging eyelids, more wrinkles, and more droopy corners of the mouth [53,54]. Alcohol consumption has also been shown to be connected to skin aging, as chronic alcohol consumption may lead to tolerance of HPA axis-activating effects and, like prolonged glucocorticoid exposure, contribute to premature or exaggerated aging [52,55].

It has also been pointed out that physical activity could on one hand improve skin health by enhancing collagen, circulation, and lowering cortisol, but on the other hand may also cause barrier damage, excess oil, and infections [56].

In addition to the variables noted above, we also included sex and UV exposure as control variables, as sex steroids and UV rays may also significantly influence skin aging patterns [57,58,59]. Additionally, given that prior research has identified a positive correlation between gray matter volume, behavioral activation, and social factors [14,60], Gray Matter GM-BHQ [13], which represents gray matter volume as a standardized score, taking into account that the degree of atrophy in gray matter varies by region, was also included as a covariate.

### 2.4. MRI Data Acquisition

MRI data were acquired on a 3-T Siemens scanner (MAGNETOM Prisma, Siemens, Munich, Germany) with a 32-channel head coil. A high-resolution structural image was obtained using a 3D T1-weighted magnetization-prepared rapid-acquisition gradient echo pulse sequence, with the following parameters: TR = 1900 ms, TE = 2.52 ms, TI = 900 ms, flip angle = 9°, matrix = 256 × 256, FOV = 256 mm, and slice thickness = 1 mm. DTI data were collected using SE-EPI with GRAPPA, aligned to the orbitomeatal line. DTI parameters included: TR = 14,100 ms, TE = 81 ms, flip angle = 90°, matrix = 114 × 114, FOV = 224 mm, slice thickness = 2 mm, a baseline image (b = 0 s/mm^2^), and 30 diffusion directions with b = 1000 s/mm^2^.

### 2.5. FA-BHQ and GM-BHQ

FA was calculated using the FA-BHQ method developed by Nemoto et al. (2017) [14]. The FA-BHQ method has been validated through its positive correlations with cognitive function [61], as well as with the personal traits of spiritual growth, happiness, and work engagement [62,63,64]. Additionally, negative correlations have been observed with anxiety [65] and fatigue [66]. The FA-BHQ has also been recognized as an international standard (H.861.1, 2018) [67] and is considered a reliable measure for assessing brain health in healthy adults, as in this population.

T1-weighted images were preprocessed and analyzed using SPM12 on MATLAB R2015b. Each MPRAGE image was segmented into gray matter (GMP), white matter (WM), and cerebrospinal fluid (CSF), with GM images normalized using the exponentiated lie algebra (DARTEL) algorithm. A modulation step was applied during preprocessing to adjust for regional volume and maintain the total GM volume prior to warping. After modulation and spatial normalization, images were smoothed with an 8 mm half-maximum (FWHM) Gaussian kernel. Intracranial volume (ICV) was calculated by summing GM, WM, and CSF volumes. To account for variations in whole-brain volume across participants, proportional GM images were generated by dividing the smoothed GM image by the ICV. These proportional GM images were then used to calculate mean and standard deviation (SD) images for all participants.

The GM-BHQ was calculated as an IQ-like measure, with a mean of 100 and an SD of 15. The formula used was 100 + 15 × (individual proportional GM−mean)/SD. Regional GM quotients were extracted using the AAL atlas and averaged to create participant-specific GM-BHQs.

DTI data were preprocessed using FSL 5.0.11. First, all diffusion images were aligned to the initial b0 image, and eddy current correction was applied to address motion and distortion. Following these corrections, FA images were generated using DTIFit. The FA images were then spatially normalized to MNI space using FLIRT and FNIRT. Mean and SD images were derived from all FA images, and individual FA quotient images were calculated using the formula 100 + 15 × (individual FA−mean)/SD. Regional FA quotients were extracted from the JHU DTI-based WM atlas and averaged across regions to create participant-specific FA-BHQs. For more details, see Nemoto et al. (2017) [14].

### 2.6. Statistical Analysis

We first conducted correlation analysis to examine the relationship between subscales of FA-BHQ and skin scale, which was measured by number of cristae cutis. A *p*-value threshold of 0.05 was adopted for statistical significance, and the Benjamini–Hochberg (BH) method was used to control for multiple comparisons. Based on the results of partial correlation, mediation analysis of specific subscales of FA-BHQ in relationship between stress and skin aging was conducted. The covariates included in both the partial correlation and mediation analyses were derived from demographic information, including sex, age, GM-BHQ, BMI, sleep status, sleep time, alcohol consumption, UV exposure, and exercise, as previously noted.

All analyses in this study were conducted using R studio (R version: 4.4.1) and SPSS version 26 (IBM Corporation, Armonk, NY, USA) with PROCESS_V4.2_beta package (models 4), which uses bootstrapping with higher power and more accurate confidence intervals, and STATA 18 SE (StataCorp, College Station, TX, USA).

## 3. Results

**Figure 4:**
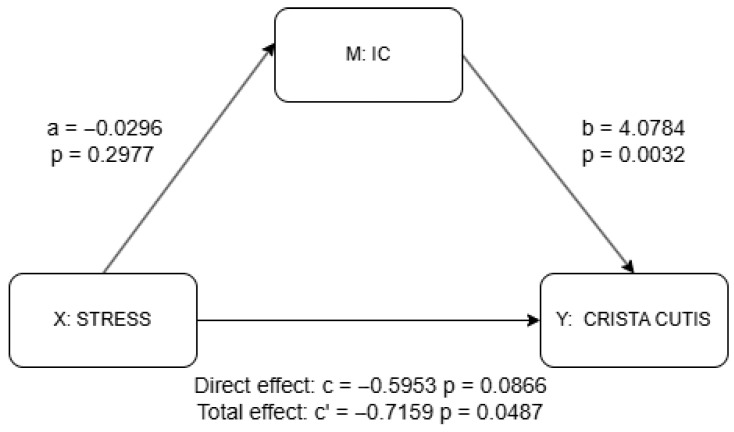
Mediation analysis results for IC using PROCESS package. a = effect of X (stress) on M (IC); b = effect of M (IC) on Y (skin scale) controlling for X (stress); c’ = total effect of X (stress) on Y (skin scale) without M (IC); c = direct effect of X (stress) on Y (skin scale) controlling for M (IC). Detailed results shown in Supplementary Materials Table S2.

**Figure 5:**
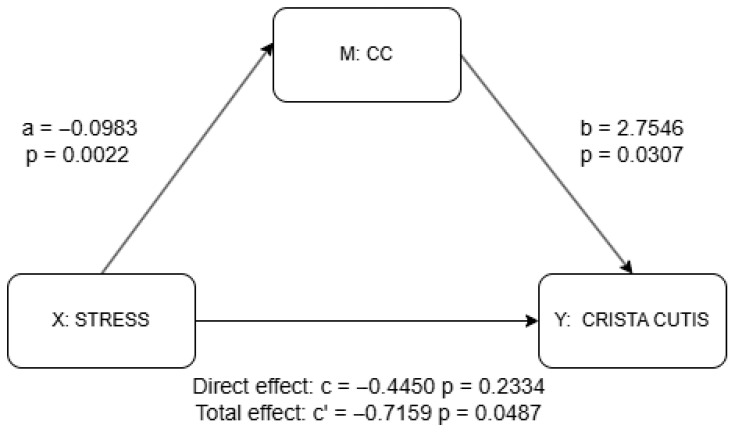
Mediation analysis results for CC using PROCESS package. a = effect of X (stress) on M (CC); b = effect of M (CC) on Y (skin scale) controlling for X (stress); c’ = total effect of X (stress) on Y (skin scale) without M (CC); c = direct effect of X (stress) on Y (skin scale) controlling for M (IC). Detailed results shown in Supplementary Materials Table S1.

The results of the correlation analysis are presented in Supplementary Materials Table S4 and Supplementary Materials Figure S1, and the results of the partial correlation analysis using control variables noted above are adjusted using the BH method to control for multiple comparisons (see Table 2 and Supplementary Materials Figure S2). Based on these findings, we selected the regions whose FA-BHQ subscales remained significant after BH correction and thus conducted an analysis to explore the mediation effect of those specific subscales of FA-BHQ in the relationship between stress and skin aging, and the results are shown in Figure 4 and Figure 5. The covariates included in both the partial correlation and mediate analyses were derived from demographic information, including sex, age, GM-BHQ, BMI, sleep status, sleep time, alcohol consumption, UV exposure, and exercise, as previously noted.

Partial correlation coefficients between the FA-BHQ subscales and skin scale, controlling for all covariates, are presented in Table 2, while scatter plots illustrating these relationships, including non-significant pairs, are shown in Supplementary Materials Figure S2. Statistically significant correlations at the 5% level were observed for the following pairs: skin scale and corpus callosum (CC) (r = 0.316, *p* = 0.001), skin scale and internal capsule (IC) (r = 0.365, *p* = 0.004), anterior corona radiata (ACR) (r = 0.241, *p* = 0.028), stress (r = −0.231, *p* = 0.036), and skin scale. However, only CC and IC pairs passed the BH method.

The results of the mediation analysis as discussed previously are shown in Figure 4 and Figure 5 as well as in Supplementary Materials Tables S1 and S2. There was no significant effect of stress on the IC (a = −0.030, *p* = 0.298), although the IC significantly predicted higher skin scale scores (b = 4.078, *p* = 0.003). While the total effect of stress on skin scale was statistically significant (c = −0.716, *p* = 0.049), the direct effect was not (c′ = −0.595, *p* = 0.087), suggesting a possible indirect pathway through the IC, though the lack of a significant a-path weakens support for mediation. In contrast, stress was significantly negatively associated with the CC (a = −0.098, *p* = 0.002), and the CC, in turn, significantly predicted skin scale (b = 2.755, *p* = 0.031). Although the total effect of stress on skin scale remained significant (c = −0.716, *p* = 0.049), the direct effect was not statistically significant (c′ = −0.445, *p* = 0.233), indicating that the CC may serve as a mediator. Taken together, only the CC provides statistically significant evidence of mediation. The absence of a significant direct effect further suggests the possibility of full mediation in this pathway.

## 4. Discussion

The speed of human skin aging is primarily influenced by photoaging; however, recent evidence also indicates that psychological stress plays a contributing role in this process. A reduction in the number of cristae cutis, often also observed as an increase in wrinkles, is one of the key expressions of skin aging. In this study, we aim to evaluate how brain WM may mediate the skin aging process driven by stress using mediation analysis. The results indicate that, across all examined regions with significant correlation with skin scale, the effect of stress on the number of cristae cutis is fully mediated by CC.

When the body experiences stress, the skin can respond through the immune system and inflammation. In the brain, stress primarily influences the skin via the hypothalamic–pituitary–adrenal (HPA) axis. Several previous studies have examined the relationship between hormones released during this process and stress-induced changes in skin condition.

This starts with neurons in the hypothalamus releasing corticotropin-releasing hormone (CRH). The CRH travels to the pituitary gland, where it attaches to CRH receptor type-1 (CRH-R1). This triggers the release of neuropeptides derived from proopiomelanocortin, such as adrenocorticotropin (ACTH). ACTH then moves through the bloodstream to the adrenal cortex, where it binds to MC2 receptors in the outer layer and prompts the production of glucocorticoids, including cortisol and corticosterone [68]. In skin, CRH and ACTH function in various ways. For example, in mast cells, CRH triggers degranulation and increases vascular permeability [9]. Also, CRH has been found to have age-related effects on the skin. In aged skin, there is an upregulation of CRH in sebaceous glands and an increased expression of CRHR1 in hair follicles and the epidermis [69]. ACTH, similarly, functions in skin via immune system. For example, ACTH stimulates IL-18 production in skin keratinocytes. IL-18 is a pro-inflammatory cytokine that enhances T-cell activity and promotes T helper type 2 (Th2) cytokines production [11].

Psychological stress is also associated with negative effects on WM microstructure through immune mediators and stress-related hormones [13,60]. The CC is the largest interhemispheric commissure in the human brain, connecting regions such as the anterior cingulate cortex and orbitofrontal cortex in both hemispheres, which are crucial for mood regulation [70]. Previous studies have found that rhesus monkeys exposed to early maternal maltreatment exhibit reduced FA in the CC, which was associated with elevated cortisol levels during infancy [71]. Additionally, stress has been shown to be related to reductions in FA in the CC, as evidenced in cases of PTSD in adolescents, as well as in patients with MDD and schizophrenia [15,16,72].

Based on the existing evidence, FA values in the CC are closely linked to exposure to psychological stress, particularly during early developmental periods [73,74,75]. Reduced FA in this white matter region may reflect disruptions in neural pathways critical for emotional processing and stress regulation—circuits often impaired in stress-related psychiatric conditions such as post-traumatic stress disorder (PTSD), major depressive disorder, bipolar disorder, and schizophrenia [76,77,78]. While it remains uncertain whether diminished white matter integrity directly contributes to dysregulation of the HPA axis, these structural alterations may indicate underlying vulnerabilities or damage associated with chronic stress or traumatic experiences. Given that activation of the HPA axis influences skin health through inflammatory and immune responses, individuals with lower FA values in CC may be more susceptible to accelerated skin aging via neuroendocrine and immunological mechanisms, or higher FA values in the CC may reflect some forms of stress resilience, as suggested in the previous literature [79].

To our knowledge, this is the first study to demonstrate a relationship between skin aging and stress and how it is associated with brain white matter microstructure. These findings suggest potential implications for the integration of skin anti-aging care, brain health promotion, and stress reduction. Reducing stress may improve mental health and may also slow aspects of skin aging. A preventive approach could combine stress management, brain-function monitoring, and evidence-based skin care to support overall well-being.

## 5. Limitation

There are three major limitations in this study.

Firstly, the sample size is relatively limited. Although the available dataset allows for statistical analysis, the restricted number of observations and cross-sectional data leaves challenges in ensuring the robustness and generalizability of the findings, particularly regarding the interpretation of full mediation. Future studies should use larger and more comprehensive panel data samples to strengthen the validity and reliability of the results.

Secondly, this study primarily provides correlational evidence regarding the potential stress-related link between WM microstructure and skin aging. However, it is important to note that, although we controlled for several related variables such as addictive habits, other confounding factors still may be involved in this relationship which were not fully accounted for in the present analysis. For example, data on hormonal status, dermatologic conditions, and medication use were not available in the current study. Therefore, causal interpretation of the results remains limited. Future research should aim to control for additional confounding variables and explore more comprehensive mechanisms underlying the association between stress, WM microstructure, and skin aging.

Another limitation of the present study lies in the use of the POMS2 TMD score. While this measure captures short-term, state-dependent emotional conditions, facial skin aging represents a long-term, cumulative biological process. Thus, potential temporal incongruity may exist between the time scale of the psychological predictor and that of the skin scale. This possible mismatch should be considered when interpreting the observed associations in the present study.

## 6. Conclusions

This study aims to examine whether white matter integrity mediates the link between psychological stress and skin aging, which is shown by the number of cristae cutis. In 92 healthy Japanese adults, we combined POLA Motion Scan skin measures with DTI-derived FA-BHQ subscales of seven selected tracts and controlled for age, sex, BMI, GM-BHQ, sleep status and time, alcohol use, exercise, and UV exposure. The mediation analysis suggested that CC integrity may serve as an important neural correlate associated with the link between stress and skin aging, which is consistent with the concept of a brain–skin axis in which reduced CC integrity is related to emotional regulation and skin aging.

These findings suggest important implications for the integration of skin anti-aging care, brain health promotion, and stress reduction. Alleviating stress may support not only mental well-being but also the prevention of skin aging, highlighting the potential to combine psychological care with dermatological care as part of a comprehensive health program. Reducing stress may improve mental health and may also slow aspects of skin aging. A preventive approach could combine stress management, brain-function monitoring, and evidence-based skin care to support overall well-being.

## Figures and Tables

**Table 1:** Functions of regions of interest in stress and emotion regulation.

Region 17.	Role	Example Associations
Fornix	Part of the limbic system; regulation of emotions by higher-order frontal cortical regions [17,18]	
Uncinate Fasciculus (UF)	Connects orbitofrontal cortex to temporal lobe; emotion regulation [18,19]	Heightened anxiety; major depressive disorder
Cingulum	Emotional processing [20,21]	Anxiety disorders; attentional bias toward negative interpersonal stimuli
Corpus Callosum (CC)	Critical interhemispheric commissure; integrates cognitive and emotional processing [22,23]	Anxiety and stress-related disorders; aggressive behavior in bipolar disorder
Internal Capsule (IC)	Convergence for thalamic radiations; emotional processing [24]	
Anterior Corona Radiata (ACR)	Connects thalamic and prefrontal regions; part of limbic-thalamo-cortical circuit; worry prediction and emotional control [25]	Bipolar I disorder
Superior Longitudinal Fasciculus (SLF)	Major association tract linking the parietal and frontal lobes; role in attention; executive function; and emotion regulation [27,28]	PTSD; OCD; depression

**Table 2:** Results of partial correlation analysis between FA-BHQ subscales, stress and skin scale controlled for GM-BHQ, age, BMI, sex, sleep status, sleep time, alcohol consumption, exercise, and UV exposure.

	Coefficient	*p*-Value	
Internal Capsule	0.365	0.001	***^‡^
Corpus Callosum	0.316	0.004	**^‡^
Fornix	0.189	0.088	
Anterior Corona Radiata	0.241	0.028	*
Cingulum	0.118	0.290	
Superior Longitudinal Fasciculus	0.153	0.167	
Stress	−0.231	0.036	*

* *p* < 0.05, ** *p* < 0.01, *** *p* < 0.001. ^‡^ *p* < 0.05 for multiple comparisons using the Benjamini and Hochberg method.

## Data Availability

The datasets generated during the current study are not publicly available but are available from the corresponding author upon reasonable request. The data presented in this study are available on request from the corresponding author due to the need to protect the privacy of participants.

## Supplementary Materials

The following supporting information can be downloaded at: https://www.mdpi.com/article/10.3390/life15111664/s1, Table S1. Detailed results of the mediation analysis of CC using the PROCESS package. Table S2. Detailed results of the mediation analysis of IC using the PROCESS package. Table S3. Descriptive Statistics of Regression Variables. Table S4. Results of correlation analysis between major variables, including subscales of FA-BHQ, control variables, stress, and skin scale. *p*-value in parentheses. Figure S1. Heatmap of correlation analysis between major variables, including subscales of FA-BHQ, control variables, stress, and skin scale. Figure S2. Scatter plot between subscales of FA-BHQ and skin scale.
