# Correction: Yuan et al. White Matter Integrity of the Corpus Callosum Mediates the Association Between Aging and Skin Condition. *Life* 2025, *15*, 1664

**DOI:** 10.3390/life16040689

**Published:** 2026-04-20

**Authors:** Daihaoyi Yuan, Keisuke Kokubun, Kiyotaka Nemoto, Yoshinori Yamakawa

**Affiliations:** 1 Graduate School of Economics, Kyoto University, Kyoto 606-8501, Japan; 2 Open Innovation Institute, Kyoto University, Kyoto 606-8501, Japan; kokubun.keisuke.6x@kyoto-u.jp (K.K.); yamakawa@bi-lab.org (Y.Y.); 3 Graduate School of Management, Kyoto University, Kyoto 606-8501, Japan; 4 Department of Medical Informatics and Management and Psychiatry, Institute of Medicine, University of Tsukuba, Tsukuba 305-8577, Japan; 5 Institute of Innovative Research, Institute of Science Tokyo, Meguro, Tokyo 226-8503, Japan; 6 ImPACT Program of Council for Science, Technology and Innovation (Cabinet Office, Government of Japan), Chiyoda, Tokyo 100-8914, Japan; 7 Office for Academic and Industrial Innovation, Kobe University, Kobe 657-0013, Japan; 8 Brain Impact, Kyoto 606-8507, Japan

In the original publication [1], there were two errors: the lack of usage rights for Figure 2 and an inaccurate description of the measurement equipment. These revisions are presented below. The authors and the Editorial Office confirm that these changes do not affect the scientific conclusions of the study. This correction was approved by the Academic Editor. The original publication has also been updated.

## Figure Correction

Figure 2 has been deleted. Accordingly, Figures 3–5 have been re-numbered to Figures 2–4, respectively.

## Main Text Correction

The sentence describing the equipment, “using Motion Scan Technology”, in the “Abstract” section has been replaced with the following sentence: “using the Smart Skin Evaluation Program (SSKEP)”.

The paragraph describing the measurement equipment in Section 2.2 (“Skin Data”), beginning “We used skin data provided by POLA Inc.” and ending “rated on a scale from 1 to 10.”, has been replaced with the following paragraph:

“In this study, skin data were obtained using the Smart Skin Evaluation Program (SSKEP) developed by Sony Corporation (1-7-1 Konan, Minato-ku, Tokyo 108-0075, Japan) [30]. SSKEP integrates proprietary CMOS image sensor technology, multi-wavelength optical illumination, and advanced image recognition and signal processing algorithms. By using skin imaging data acquired across the spectral range from near-ultraviolet to near-infrared wavelengths, including the visible spectrum, the system acquires layered images that capture surface and subsurface skin information, which are analyzed at the pixel level to quantify indices such as skin texture, pore characteristics, pigmentation and redness, tone, and sebum. SSKEP enables objective and reproducible quantification of multiple skin attributes, making it suitable for empirical research. Recent studies by the same research group further demonstrate that near-infrared and hyperspectral texture features are systematically associated with biophysical skin properties, including skin elasticity, supporting the validity of extracting quantitative texture-related indices from multi-band skin images [31,32].”

The sentence describing the equipment, “POLA Motion Scan skin measures”, in the Section 6 “Conclusions” has been replaced with the following sentence: “an established skin evaluation method”.

## References Correction

The reference “[31] Kurosumi, M.; Mizukoshi, K.; Hongo, M.; Kamachi, M.G. The Effect of Observation Angles on Facial Age Perceptions: A Case Study of Japanese Women. *PLoS ONE* **2022**, *17*, e0279339.” has been replaced with the following reference: “[31] Kim, J.; Lee, O. Correlation Analysis Between Texture Features and Elasticity of Skin Hyperspectral Images in the Near-Infrared Band. *Skin Res. Technol*. **2024**, *30*, e13654. https://doi.org/10.1111/srt.13654.”

The reference “[32] Kurosumi, M.; Yabuzaki, J.; Kuribayashi, M.; Mizukoshi, K. Age-related Changes in Cheek Skin Movement: A Case Study of Japanese Women. *Skin Res. Technol*. **2024**, *30*, e13768.” has been replaced with the following reference: “[32] Kim, J.; Lee, O. Hyperspectral Image Wavelength Band and Texture Characteristics Related to Elasticity Values for Each Body Part for Non-Invasive Skin Elasticity Analysis. *Biomed. Opt. Express* **2025**, *16*, 3573–3588. https://doi.org/10.1364/BOE.571166.”
