# Supplementary material for: White Matter Integrity of the Corpus Callosum Mediates the Association Between Aging and Skin Condition
Source: Life (Basel). 2025 Oct 24;15(11):1664. doi: 10.3390/life15111664 (PMC12653096; doi:10.3390/life15111664)
Supplement: Supplementary file 1 [file life-15-01664-s001.zip › life-3907589-supplementary.pdf]

life

MDPI

Supplementary Materials

Table S1. Detailed results of the mediation analysis of CC using the PROCESS package. Block 1 presents the regression of stress on corpus callosum (CC); Block 2 reports the regression of stress on cristae cutis (skin scale), including CC as a mediator regression. Block 3 reports the regression of stress on skin scale without CC as a mediator. All models include the same set of control variables as noted above.

OUTCOME VARIABLE:						
CC						
Model Summary						
R	R-sq	MSE	F	df1	df2	*p*
0.5043	0.2544	20.2205	2.7632	10.0000	81.0000	0.0055
Model						
Variable	Coeff.	Std. Error	t-Statistic	*p*-Value	LLCI	ULCI
Constant	82.3802	14.4822	5.6884	0.0000	53.5650	111.1953
Stress	−0.0983	0.0310	−3.1703	0.0022	−0.1601	−0.0366
Age	−0.0573	0.0757	−0.7576	0.4509	−0.2078	0.0932
Sex	−2.6736	1.2565	−2.1278	0.0364	−5.1735	−0.1736
GM-BHQ	0.2139	0.1070	1.9996	0.0489	0.0011	0.4268
Exercise	0.0006	0.0003	1.7537	0.0833	−0.0001	0.0013
BMI	0.0454	0.1459	0.3109	0.7567	−0.2450	0.3357
UV	−0.4498	0.6065	−0.7415	0.4605	−1.6566	0.7570
Sleep Status	2.2329	1.0288	2.1704	0.0329	0.1859	4.2799
Alcohol Consumption	0.0116	0.2683	0.0431	0.9657	−0.5222	0.5453
Sleep Time	−2.3973	1.3520	−1.7732	0.0800	−5.0873	0.2927
OUTCOME VARIABLE:						
Skin Scale						
Model Summary						
R	R-sq	MSE	F	df1	df2	*p*
0.5082	0.2582	2567.5799	2.5317	11	80	0.0086
Model						
Variable	Coeff.	Std. Error	t-Statistic	*p*-Value	LLCI	ULCI
Constant	526.0429	193.056	2.7248	0.0079	141.848	910.2379
Stress	−0.445	0.3706	−1.2007	0.2334	−1.1825	0.2925
CC	2.7546	1.2521	2.2001	0.0307	0.2629	5.2463
Age	−2.0385	0.8555	−2.3829	0.0195	−3.741	−0.3361
Sex	19.1054	14.5488	1.3132	0.1929	−9.8477	48.0585
GM-BHQ	−2.9901	1.2349	−2.4214	0.0177	−5.4477	−0.5326
Exercise	−0.0054	0.0039	−1.3895	0.1685	−0.0133	0.0024
BMI	−1.6416	1.6452	−0.9978	0.3214	−4.9157	1.6325
UV	−13.2374	6.8578	−1.9303	0.0571	−26.8849	0.4101
Sleep Status	15.5623	11.9253	1.305	0.1956	−8.1699	39.2945
Alcohol Consumption	−1.7333	3.0229	−0.5734	0.568	−7.7491	4.2825
Sleep Time	−34.4741	15.5277	−2.2202	0.0292	−65.3753	−3.573
OUTCOME VARIABLE:						
Skin Scale						
Model Summary						
R	R-sq	MSE	F	df1	df2	*p*

0.4619	0.2133	2689.3106	2.1967	10	81	0.026
Model						
Variable	Coeff.	Std. Error	t-Statistic	*p*-Value	LLCI	ULCI
Constant	752.9669	167.0164	4.5083	0	420.6555	1085.2782
Stress	−0.7159	0.3577	−2.0011	0.0487	−1.4277	−0.0041
Age	−2.1964	0.8724	−2.5176	0.0138	−3.9323	−0.4605
Sex	11.7408	14.4902	0.8103	0.4202	−17.0902	40.5718
GM-BHQ	−2.4009	1.2338	−1.946	0.0551	−4.8557	0.0539
Exercise	−0.0038	0.0039	−0.9643	0.3378	−0.0116	0.004
BMI	−1.5166	1.6828	−0.9013	0.3701	−4.8648	1.8316
UV	−14.4763	6.9948	−2.0696	0.0417	−28.3937	−0.5589
Sleep Status	21.713	11.8646	1.8301	0.0709	−1.8939	45.3199
Alcohol Consumption	−1.7014	3.0937	−0.55	0.5839	−7.8569	4.4541
Sleep Time	−41.0778	15.5918	−2.6346	0.0101	−72.1006	−10.055

Table S2. Detailed results of the mediation analysis of IC using the PROCESS package. Block 1 presents the regression of stress on internal capsule (IC); Block 2 reports the regression of stress on cristae cutis (skin scale), including IC as a mediator regression. Block 3 reports the regression of stress on skin scale without IC as a mediator. All models include the same set of control variables as noted above.

OUTCOME VARIABLE:						
IC						
Model Summary						
R	R-sq	MSE	F	df1	df2	*p*
0.309	0.0955	16.7294	0.8553	10.0000	81.0000	0.5778
Model						
Variable	Coeff.	Std. Error	t-Statistic	*p*-Value	LLCI	ULCI
Constant	101.7298	13.1728	7.7227	0.0000	75.5199	127.9397
Stress	−0.0296	0.0282	−1.0482	0.2977	−0.0857	0.0266
Age	−0.0416	0.0688	−0.6050	0.5469	−0.1785	0.0953
Sex	−1.5133	1.1429	−1.3242	0.1892	−3.7873	0.7606
GM-BHQ	0.0293	0.0973	0.3016	0.7637	−0.1643	0.2230
Exercise	0.0002	0.0003	0.6349	0.5273	−0.0004	0.0008
BMI	−0.0094	0.1327	−0.0705	0.9440	−0.2734	0.2547
UV	−0.3486	0.5517	−0.6319	0.5292	−1.4463	0.7491
Sleep Status	0.7422	0.9358	0.7932	0.4300	−1.1197	2.6042
Alcohol Consumption	0.2795	0.2440	1.1453	0.2555	−0.2060	0.7650
Sleep Time	−2.0613	1.2297	−1.6762	0.0975	−4.5082	0.3855
OUTCOME VARIABLE:						
Skin Scale						
Model Summary						
R	R-sq	MSE	F	df1	df2	*p*
0.5429	0.2947	2441.1849	3.0393	11	80	0.0019
Model						
Variable	Coeff.	Std. Error	t-Statistic	*p*-Value	LLCI	ULCI
Constant	338.0744	209.677	1.6124	0.1108	−79.1975	755.3463
Stress	−0.5953	0.3431	−1.7348	0.0866	−1.2781	0.0876
CC	4.0784	1.3422	3.0386	0.0032	1.4073	6.7494
Age	−2.0267	0.8331	−2.4327	0.0172	−3.6846	−0.3687
Sex	17.9128	13.9542	1.2837	0.203	−9.857	45.6826
GM-BHQ	−2.5206	1.1761	−2.1431	0.0351	−4.8611	−0.18

Exercise	−0.0046	0.0038	−1.2235	0.2247	−0.0121	0.0029
BMI	−1.4785	1.6033	−0.9221	0.3592	−4.6692	1.7122
UV	−13.0546	6.6807	−1.9541	0.0542	−26.3496	0.2405
Sleep Status	18.6858	11.3478	1.6466	0.1036	−3.8971	41.2688
Alcohol Consumption	−2.8411	2.9713	−0.9562	0.3419	−8.7542	3.072
Sleep Time	−32.6708	15.1105	−2.1621	0.0336	−62.7418	−2.5998
OUTCOME VARIABLE:						
Skin Scale						
Model Summary						
R	R-sq	MSE	F	df1	df2	*p*
0.4619	0.2133	2689.3106	2.1967	10	81	0.026
Model						
Variable	Coeff.	Std. Error	t-Statistic	*p*-Value	LLCI	ULCI
Constant	752.9669	167.0164	4.5083	0	420.6555	1085.2782
Stress	−0.7159	0.3577	−2.0011	0.0487	−1.4277	−0.0041
Age	−2.1964	0.8724	−2.5176	0.0138	−3.9323	−0.4605
Sex	11.7408	14.4902	0.8103	0.4202	−17.0902	40.5718
GM-BHQ	−2.4009	1.2338	−1.946	0.0551	−4.8557	0.0539
Exercise	−0.0038	0.0039	−0.9643	0.3378	−0.0116	0.004
BMI	−1.5166	1.6828	−0.9013	0.3701	−4.8648	1.8316
UV	−14.4763	6.9948	−2.0696	0.0417	−28.3937	−0.5589
Sleep Status	21.713	11.8646	1.8301	0.0709	−1.8939	45.3199
Alcohol Consumption	−1.7014	3.0937	−0.55	0.5839	−7.8569	4.4541
Sleep Time	−41.0778	15.5918	−2.6346	0.0101	−72.1006	−10.055

Table S3. Descriptive Statistics of Regression Variables.

**Descriptive Statistics**				
	Mean	Sd	Min	Max
Number Of Crista Cutis	352.9022	55.16311	192	500
Corpus Callosum	100.0689	4.913093	84.06319	111.697
Fornix	100.4452	5.374431	80.53237	111.7703
Internal Capsule	100.6391	4.057506	87.7814	108.4317
Anterior Corona Radiata	103.4265	6.733176	84.78752	117.097
Cingulum	99.65945	4.255254	90.76009	108.6531
Superior Longitudinal Fasciculus	100.6088	4.599986	90.68784	109.3068
Uncinate Fasciculus	99.40788	6.879379	84.03642	118.0696
GM-BHQ	100.7667	8.145894	77.5795	118.336
BMI(kg/m^2)	23.86525	3.67775	17.43285	37.55102
Sex	1.326087	0.4713482	1	2
Sleep Status	2.282609	0.7311903	1	4
Alcohol Consumption	3.043478	1.880572	1	7
UV Exposure	3.152174	0.8636084	1	4
Sleep Time	1.576087	0.7298004	0	3
Exercise (MET-minute/week)	920.1522	1480.47	0	9702
Age(years)	45.07609	9.92193	22	62
Stress	19.72	18.279	−10	73

Table S4. Results of correlation analysis between major variables, including subscales of FA-BHQ, control variables, stress, and skin scale. *p*-value in parentheses.

Variable	Number Of Crista Cutis	Stress	Age	Bmi	Sex	GM-BHQ	Uv	Alcohol Consumption	Sleep Status
Number Of Crista Cutis	1.000	-0.033 (0.755)	-0.116 (0.269)	-0.009 (0.933)	0.042 (0.689)	-0.021 (0.839)	-0.205* (0.050)	-0.071 (0.499)	-0.019 (0.860)
Stress	-0.033 (0.755)	1.000	-0.074 (0.484)	0.067 (0.523)	0.008 (0.938)	-0.053 (0.617)	-0.219* (0.036)	-0.203 (0.052)	0.354***‡ (0.000)
Age	-0.116 (0.269)	-0.074 (0.484)	1.000	0.103 (0.328)	-0.057 (0.589)	'-0.692***‡ (0.000)	-0.237* (0.023)	-0.016 (0.879)	0.073 (0.491)
BMI	-0.009 (0.933)	0.067 (0.523)	0.103 (0.328)	1.000	'-0.286**‡ (0.006)	'-0.353***‡ (0.001)	-0.012 (0.909)	-0.050 (0.635)	0.130 (0.217)
Sex	0.042 (0.689)	0.008 (0.938)	-0.057 (0.589)	'-0.286**‡ (0.006)	1.000	0.452***‡ (0.000)	-0.150 (0.153)	0.033 (0.752)	0.080 (0.446)
GM-BHQ	-0.021 (0.839)	-0.053 (0.617)	'-0.692***‡ (0.000)	-0.353***‡ (0.000)	0.452***‡ (0.000)	1.000	0.160 (0.127)	0.093 (0.379)	-0.010 (0.928)
Uv	-0.205* (0.050)	-0.219* (0.036)	-0.237* (0.023)	-0.012 (0.909)	-0.150 (0.153)	0.160 (0.127)	1.000	0.192 (0.067)	-0.208** (0.047)
Alcohol Consumption	-0.071 (0.499)	-0.203 (0.052)	-0.016 (0.879)	-0.050 (0.635)	0.033 (0.752)	0.093 (0.379)	0.192 (0.067)	1.000	0.119 (0.259)
Sleep Status	-0.019 (0.860)	0.354***‡ (0.000)	0.073 (0.491)	0.130 (0.217)	0.080 (0.446)	-0.010 (0.928)	0.208* (0.047)	0.119 (0.259)	1.000
Sleep Time	-0.235* (0.024)	-0.001 (0.995)	0.087 (0.407)	0.008 (0.939)	0.030 (0.775)	-0.022 (0.839)	-0.043 (0.684)	0.106 (0.316)	0.645***‡ (0.000)
Exercise	-0.135 (0.198)	0.013 (0.905)	0.109 (0.302)	-0.124 (0.238)	-0.084 (0.426)	-0.182 (0.082)	-0.015 (0.888)	-0.054 (0.609)	-0.088 (0.405)
Corpus Callosum	0.233* (0.026)	-0.239* (0.022)	'-0.275**‡ (0.008)	-0.035 (0.742)	-0.086 (0.413)	0.273**‡ (0.009)	0.063 (0.550)	0.092 (0.384)	0.018 (0.866)
Fornix	0.171 (0.103)	-0.186 (0.076)	-0.526***‡ (0.000)	0.030 (0.780)	0.048 (0.653)	0.456***‡ (0.000)	0.068 (0.519)	-0.085 (0.421)	-0.083 (0.429)
Internal Capsule	0.333***‡ (0.001)	-0.092 (0.381)	-0.113 (0.284)	0.002 (0.982)	-0.130 (0.218)	0.040 (0.703)	0.022 (0.834)	0.129 (0.220)	-0.077 (0.467)
Anterior Corona Radiata	0.212* (0.043)	-0.162 (0.124)	-0.196 (0.061)	-0.086 (0.415)	-0.028 (0.794)	0.222* (0.033)	-0.033 (0.754)	0.043 (0.684)	0.036 (0.733)
Cingulum	0.044 (0.676)	-0.186 (0.075)	0.004 (0.970)	-0.030 (0.774)	-0.220* (0.035)	-0.076 (0.470)	0.051 (0.629)	-0.022 (0.837)	-0.155 (0.140)
Superior Longitudinal Fasciculus	0.102 (0.333)	-0.028 (0.788)	0.080 (0.450)	-0.079 (0.454)	-0.140 (0.182)	-0.069 (0.512)	-0.016 (0.883)	0.080 (0.448)	0.051 (0.632)
Uncinate Fasciculus	0.012 (0.911)	-0.167 (0.111)	0.082 (0.437)	0.060 (0.573)	-0.148 (0.159)	-0.143 (0.175)	-0.131 (0.212)	0.042 (0.689)	-0.204 (0.051)

Variable	Sleep Time	Exercise	Corpus Callosum	Fornix	Internalcapsule	Anterior Corona Radiata	Cingulum	Superior Longitudinal Fasciculus	Uncinate Fasciculus
Number Of Crista Cutis	-0.235* (0.024)	-0.135 (0.198)	0.233*‡ (0.026)	0.171 (0.103)	0.333**‡ (0.001)	0.212* (0.043)	0.044 (0.676)	0.102 (0.333)	0.012 (0.911)
Stress	-0.001 (0.995)	-0.013 (0.905)	-0.239*‡ (0.022)	-0.186 (0.076)	-0.092 (0.381)	-0.162 (0.124)	-0.186 (0.075)	-0.028 (0.788)	-0.167 (0.111)
Age	0.087 (0.407)	0.109 (0.302)	'-0.275**‡ (0.008)	'-0.526***‡ (0.000)	-0.113 (0.284)	-0.196 (0.061)	0.004 (0.970)	0.080 (0.450)	0.082 (0.437)
BMI	0.008 (0.939)	-0.124 (0.238)	-0.035 (0.742)	0.030 (0.780)	0.002 (0.982)	-0.086 (0.415)	-0.030 (0.774)	-0.079 (0.454)	0.060 (0.573)
Sex	0.030 (0.775)	-0.084 (0.426)	-0.086 (0.413)	0.048 (0.653)	-0.130 (0.218)	-0.028 (0.794)	-0.0077	-0.140 (0.182)	-0.148 (0.159)
GM-BHQ	0.022 (0.839)	-0.182 (0.082)	0.273**‡ (0.009)	0.456***‡ (0.000)	0.040 (0.703)	0.222* (0.033)	-0.076 (0.470)	-0.069 (0.512)	-0.143 (0.175)
Uv	-0.043 (0.684)	-0.015 (0.888)	0.063 (0.550)	0.068 (0.519)	0.022 (0.834)	-0.033 (0.754)	0.051 (0.629)	-0.016 (0.883)	-0.131 (0.212)
Alcohol Consumption	0.106 (0.316)	-0.054 (0.609)	0.092 (0.384)	-0.085 (0.421)	0.129 (0.220)	0.043 (0.684)	-0.022 (0.837)	0.080 (0.448)	0.042 (0.689)
Sleep Status	0.645***‡ (0.000)	-0.088 (0.405)	0.018 (0.866)	-0.083 (0.429)	-0.077 (0.467)	0.036 (0.733)	-0.155 (0.140)	0.051 (0.632)	-0.204 (0.051)
Sleep Time	1.000	0.100 (0.343)	0.018 (0.861)	0.011 (0.917)	-0.158 (0.133)	-0.012 (0.907)	-0.017 (0.875)	-0.040 (0.704)	-0.195 (0.063)
Exercise	0.100 (0.343)	1.000	0.063 (0.550)	-0.110 (0.297)	0.021 (0.842)	-0.007 (0.950)	0.147 (0.163)	0.075 (0.478)	0.093 (0.379)
Corpus Callosum	-0.018 (0.861)	0.063 (0.550)	1.000	0.611***‡ (0.000)	0.663***‡ (0.000)	0.832***‡ (0.000)	0.535***‡ (0.000)	0.487***‡ (0.000)	0.399***‡ (0.000)
Fornix	0.011 (0.917)	-0.110 (0.297)	0.611***‡ (0.000)	1.000	0.274**‡ (0.008)	0.603***‡ (0.000)	0.399***‡ (0.000)	0.124 (0.238)	0.174 (0.098)
Internal Capsule	-0.158 (0.133)	0.021 (0.842)	0.663***‡ (0.000)	0.274***‡ (0.008)	1.000	0.612***‡ (0.000)	0.599***‡ (0.000)	0.718***‡ (0.000)	0.476***‡ (0.000)
Anterior Corona Radiata	-0.012 (0.907)	-0.007 (0.950)	0.832***‡ (0.000)	0.603***‡ (0.000)	0.612***‡ (0.000)	1.000	0.558***‡ (0.000)	0.514***‡ (0.000)	0.427***‡ (0.000)
Cingulum	-0.017 (0.875)	0.147 (0.163)	0.535***‡ (0.000)	0.399***‡ (0.000)	0.599***‡ (0.000)	0.558***‡ (0.000)	1.000	0.598***‡ (0.000)	0.450***‡ (0.000)
Superior Longitudinal Fasciculus	-0.040 (0.704)	0.075 (0.478)	0.487***‡ (0.000)	0.124 (0.238)	0.718***‡ (0.000)	0.514***‡ (0.000)	0.598***‡ (0.000)	1.000	0.436***‡ (0.000)
Uncinate Fasciculus	-0.195 (0.063)	0.093 (0.379)	0.399***‡ (0.000)	0.174 (0.098)	0.476***‡ (0.000)	0.427***‡ (0.000)	0.450***‡ (0.000)	0.436***‡ (0.000)	1

* $p < 0.05$, ** $p < 0.01$, *** $p < 0.001$. $p < 0.05$ for multiple comparisons using the Benjamini and Hochberg method.

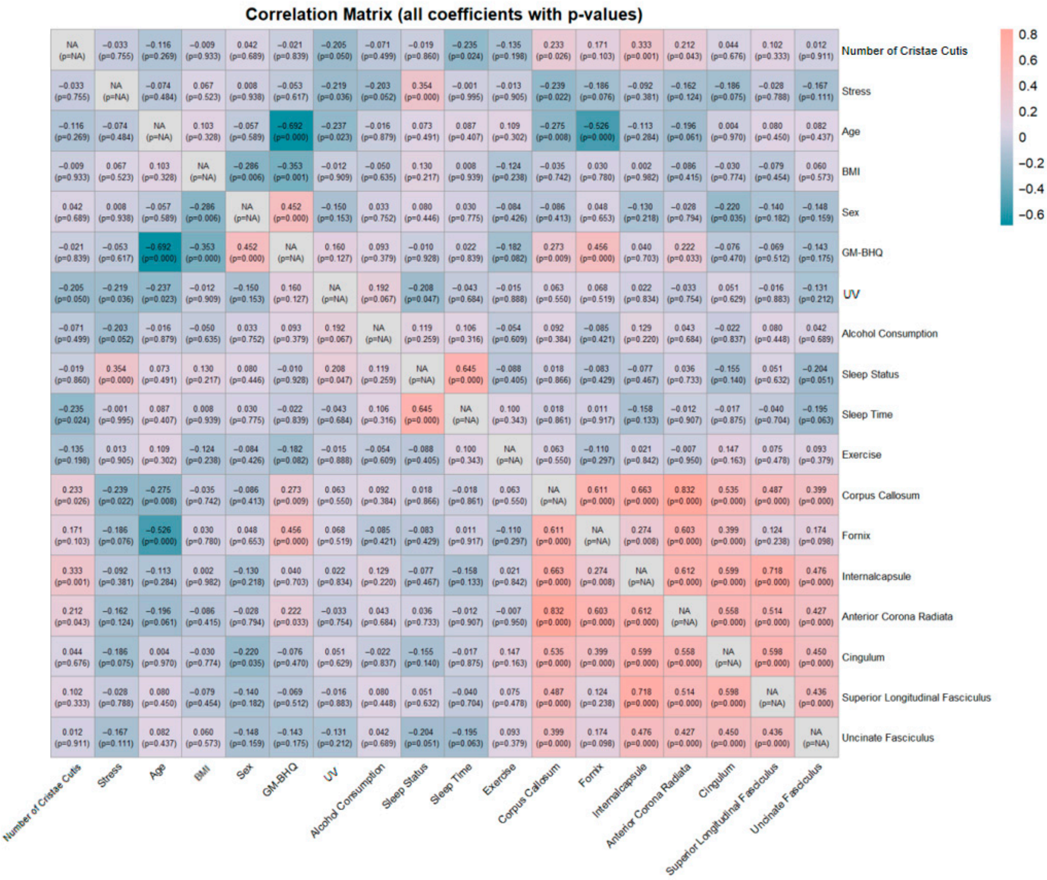

Figure S1. Heatmap of correlation analysis between major variables, including subscales of FA-BHQ, control variables, stress, and skin scale. *p*-value in parentheses.

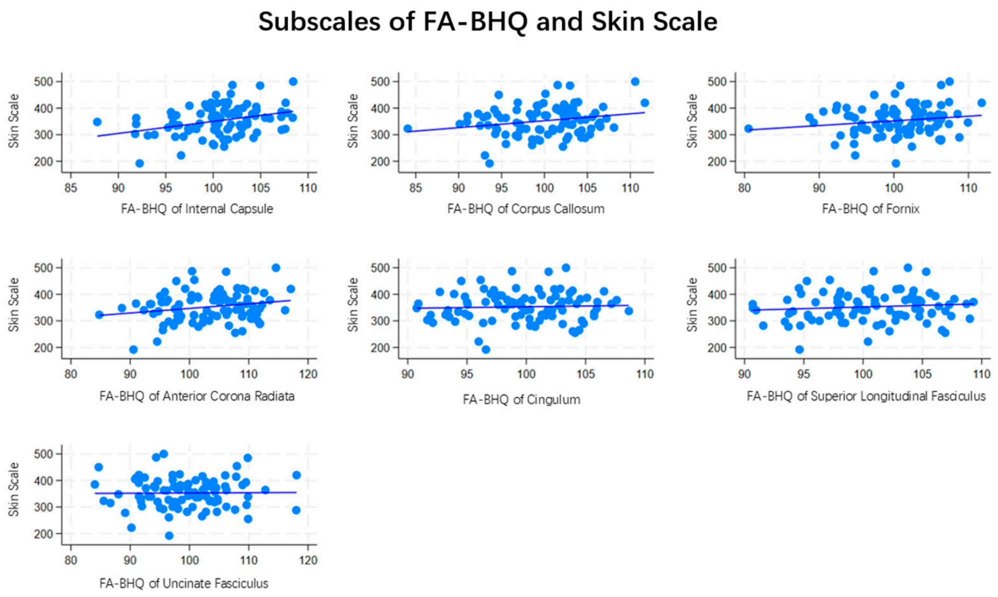

Figure S2. Scatter plot between subscales of FA-BHQ and skin scale.
